# Ivermectin-Loaded Mesoporous Silica and Polymeric Nanocapsules: Impact on Drug Loading, In Vitro Solubility Enhancement, and Release Performance

**DOI:** 10.3390/pharmaceutics16030325

**Published:** 2024-02-26

**Authors:** Maiara Callegaro Velho, Nadine Lysyk Funk, Monique Deon, Edilson Valmir Benvenutti, Silvio Buchner, Ruth Hinrichs, Diogo André Pilger, Ruy Carlos Ruver Beck

**Affiliations:** 1 Programa de Pós-Graduação em Ciências Farmacêuticas, Universidade Federal do Rio Grande do Sul (UFRGS), Porto Alegre 90610-000, RS, Brazil; maiaracvelho@hotmail.com (M.C.V.); nadine.lysyk@ufrgs.br (N.L.F.); 2 Laboratório de Nanocarreadores e Impressão 3D em Tecnologia Farmacêutica (Nano3D), Faculdade de Farmácia—UFRGS, Av. Ipiranga, 2752, 4° Andar, Porto Alegre 90610-000, RS, Brazil; 3 Programa de Pós-Graduação em Biociências, Universidade Federal de Ciências da Saúde de Porto Alegre (UFCSPA), Porto Alegre 90050-170, RS, Brazil; 4 Laboratório de Sólidos e Superfícies, Instituto de Química—UFRGS, Porto Alegre 90650-001, RS, Brazil; benvenutti@ufrgs.br; 5 Laboratório de Altas Pressões e Materiais Avançados (LAPMA), Instituto de Física—UFRGS, Porto Alegre 91501-970, RS, Brazil; silvio.buchner@ufrgs.br; 6 Instituto de Geociências (IGEO)—UFRGS, Porto Alegre 90650-001, RS, Brazil; ruth.hinrichs@ufrgs.br; 7 Laboratório de Análises Bioquímicas e Citológicas, Faculdade de Farmácia—UFRGS, Porto Alegre 90610-000, RS, Brazil; diogo.pilger@ufrgs.br

**Keywords:** enhanced solubility, ivermectin, nanoparticles, silica

## Abstract

Ivermectin (IVM), a widely used drug for parasitic infections, faces formulation and application challenges due to its poor water solubility and limited bioavailability. Pondering the impact of IVM’s high partition coefficient value (log P) on its drug release performance, it is relevant to explore whether IVM nanoencapsulation in organic or inorganic nanoparticles would afford comparable enhanced aqueous solubility. To date, the use of inorganic nanoparticles remains an unexplored approach for delivering IVM. Therefore, here we loaded IVM in mesoporous silica particles (IVM-MCM), as inorganic nanomaterial, and in well-known poly(ε-caprolactone) nanocapsules (IVM-NC). IVM-MCM had a well-organized hexagonal mesoporous structure, reduced surface area, and high drug loading of 10% *w*/*w*. IVM-NC had a nanometric mean size (196 nm), high encapsulation efficiency (100%), physicochemical stability as an aqueous dispersion, and drug loading of 0.1% *w*/*w*. Despite differing characteristics, both nanoencapsulated forms enhance IVM’s aqueous intrinsic solubility compared to a crystalline IVM: after 72 h, IVM-MCM and IVM-NC achieve 72% and 78% releases through a dialysis bag, whereas crystalline IVM dispersion achieves only 40% drug diffusion. These results show distinct controlled release profiles, where IVM-NC provides a deeper sustained controlled release over the whole experiment compared to the inorganic nanomaterial (IVM-MCM). Discussing differences, including drug loading and release kinetics, is crucial for optimizing IVM’s therapeutic performance. The study design, combined with administration route plans and safety considerations for humans and animals, may expedite the rational optimization of IVM nanoformulations for swift clinical translation.

## 1. Introduction

Ivermectin (IVM) is a broad-spectrum antiparasitic drug that has been used for more than three decades to treat a variety of parasitic infections in humans and animals [1]. Discovered in the 1970s by Japanese scientist Satoshi Ōmura, IVM was initially proposed for the treatment of parasitic diseases in animals, being introduced to the veterinary market in the early 1980s, and shortly thereafter approved for use in humans in 1987 by the U.S. Food and Drug Administration (FDA) as an oral treatment for onchocerciasis (*Onchocerca volvulus*) [2].

In the past few years, a wide range of biological activities has been described for IVM, arousing the attention of medical and pharmaceutical researchers for its potential for drug repositioning. A notable increase in interest in IVM occurred in 2020, due to the coronavirus disease (COVID-19) pandemic and the evidence of IVM as a potential in vitro inhibitor of the viral replication of coronavirus 2 (SARS-CoV-2) [3]. Nevertheless, the demonstration of IVM’s antiviral effect was not unprecedented. Previous studies had already reported its potential as an inhibitor in flavivirus replication [4], HIV-1 virus [5], dengue, and Zika [6,7]. Studies have also suggested IVM application as an antibacterial agent [8,9,10] and potential insecticide [11,12,13]. Furthermore, the anti-inflammatory [14,15,16] and anticancer properties of IVM have been recognized [17,18,19]. In the last eight years, preclinical trials were conducted with promising results against breast adenocarcinoma, glioma, colon cancer, kidney cancer, leukemia, and melanoma [17,18,19].

**Figure 1:**
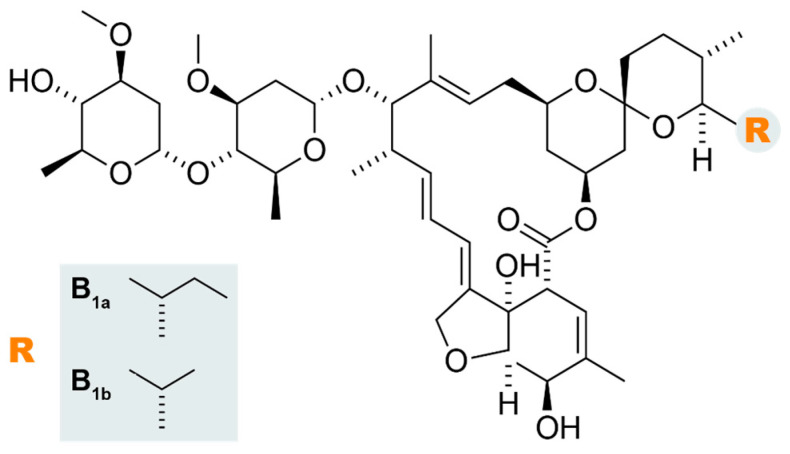
Representation of the molecular structure of ivermectin.

IVM is derived from avermectin, an isolated class of fermentation products of *Streptomyces avermitilis* [20]. Its structure contains a 16-member ring (Figure 1), consisting of a synthetic mixture of two homologs containing approximately 80% 22,23-dihydroavermectin-B1a (molecular weight, 875.10 g/mol) and 20% 22,23-dihydroavermectin-B1b (molecular weight, 861.07 g/mol) [19]. Due to its chemical structure, which contains a lactone nucleus of 16 carbon atoms and only two hydroxyl (OH) groups at its ends, IVM has poor water solubility (~4 μg/mL or ~5 μM in pure water) [21].

IVM has a partition coefficient (log P) value of 5.83 [22], indicating its strong affinity for lipids and limited solubility in water. According to the Biopharmaceutics Drug Classification System (BCS), IVM falls into Class II, characterized by low solubility and high permeability [23]. This means that IVM can effectively cross biological membranes but faces challenges dissolving in aqueous environments such as body fluids and is thus an honest candidate for studies to overcome its poor aqueous solubility. The limited solubility of IVM in body fluids can negatively impact its dissolution rate and bioavailability.

Knowing the Log P and BCS class of IVM is essential to set up some strategies to enhance its therapeutic efficacy against parasitic infections and other potential therapeutic applications. It enables the accurate prediction of in vivo performance and facilitates the development of optimized pharmaceutical formulations and administration strategies. Although IVM has shown great pharmacological potential for treating parasitic infections, its biopharmaceutical limitations restrict its use in other applications. Usually, the recommended dose of IVM as an antiparasitic is 0.2 mg/kg for humans and non-human species. However, for new therapeutic applications, new dosage regimens must be considered. In this sense, the improvement of its biopharmaceutical characteristics, such as aqueous solubility, is crucial. The enhancement of drug dissolution in biological fluids increases the absorption rate and, consequently, positively affects the beginning of the biological effect and drug exposure time, favoring the achievement of an effective plasma dose [1].

In the last decade, there has been a significant increase in studies focusing on drug delivery techniques that can improve the solubility of BCS Class II drugs [24]. This is because a substantial proportion of drugs with market approval (around 40%) and most molecules in the discovery pipeline (almost 90%) are poorly soluble in water [25].

Numerous strategies have been proposed to improve the dissolution rate of drugs such as IVM, including the application of nanotechnology-based delivery systems. These systems often incorporate a variety of nanomaterials, frequently utilizing hybrid lipid-based and synthetic polymer matrices. IVM-loaded nanoparticles were primarily designed for antiparasitic applications, emphasizing key optimization factors such as safeguarding against degradation, enhancing permeability [13,26], ensuring physicochemical stability [27], controlled drug release [7,28], enhancing cutaneous penetration [29], and precise site-specific delivery [30]. It is worth underscoring that despite the advancements, a comprehensive characterization and detailed understanding of targeted delivery mechanisms and drug release profiles are still lacking for many of these nanomaterials.

Nanostructured carriers have unique properties that can improve drug solubility, enhancing therapeutic efficacy and ensuring greater precision dosing. These properties are obtained via pharmacokinetic modulation [26]. Organic and inorganic nanomaterials can be used to achieve such effects and, while organic nanomaterials stand for better biocompatibility and biodegradability [31], inorganic nanocarriers present higher stability and a higher loading capacity.

Among the organic nanocarriers, our research group has been studying the behavior of polymeric nanocapsules, a vesicular system consisting of an oily nucleus surrounded by a polymer shell and a layer of hydrophilic surfactant, where the drug can be either dissolved in the core or adsorbed on the polymeric wall [32]. The presence of an oily core in nanocapsules favors the high encapsulation efficiency of hydrophobic drugs, such as IVM. Nanocapsule structure not only stabilizes the drug in an aqueous medium but, in some cases, also enables targeted delivery of the drug to specific cells or tissues [33]. In this regard, poly(e-caprolactone) (PCL) nanocapsules are versatile formulations available in liquid, semi-solid, or solid forms. PCL, a semicrystalline aliphatic polyester, is capable of controlling drug release and improving stability in different media. Its biodegradability and biocompatibility make it suitable for medical devices, tissue engineering, and drug carriers [32]. 

In parallel, our research group has been studying drug encapsulation in mesoporous silica nanoparticles (MSNs) as inorganic drug carriers, which have gained notorious prominence in recent years [34]. However, to the best of our knowledge, MSNs remain an unexplored approach for delivering IVM. MSNs hold uniform and nanometric-sized pores with dimensions ranging from 2 nm to 50 nm, known as mesopores. These mesopores can be organized in cubic, hexagonal, or lamellar structures, providing space for drug accommodation and enabling surface functionalization [35]. 

Among the distinct types of MSNs, MCM-41 is well known for its 2D porous hexagonal structure and narrow distribution of mesopores with a large volume and high surface area. The highly porous structure enables the adsorption and transport of large amounts of molecules or drugs [36] and a confinement effect of the molecules in nanometric pores can stabilize the loaded drug in its amorphous state, which is associated with an increase in the dissolution/release rate and bioavailability [37,38]. 

Given the distinctive attributes of the aforementioned nanostructured systems, this study aims to discern how the encapsulation of IVM within inorganic nanocarriers (mesoporous silica particles) and organic nanocarriers (poly-ε-caprolactone nanocapsules) influences its intrinsic solubility and modulates its in vitro release profile. The significant contribution of our research lies in the meticulous and reasoned examination of these two disparate nanostructured systems, both organic and inorganic. This investigation represents a pioneering effort to systematically evaluate their potential as promising approaches for augmenting the efficacy of IVM delivery. Moreover, this assessment was conducted by considering the diverse therapeutic opportunities that IVM presents, thereby underscoring the broad implications and versatility of our findings in advancing drug delivery strategies.

## 2. Materials and Methods

### 2.1. Materials

Ivermectin, poly(ε-caprolactone), nanostructured silica type MCM-41 (hexagonal), and dialysis bag membranes were acquired from Sigma-Aldrich (São Paulo, Brazil). Medium-chain triglycerides and polysorbate 80 (Tween 80^®^) were obtained from Delaware (Porto Alegre, Brazil) and Henrifarma (São Paulo, Brazil), respectively. Acetone was purchased from Neon Comercial LTDA (São Paulo, Brazil), and analytical (HPLC) grade Acetonitrile and Methanol were purchased from Merck (Darmstadt, Germany).

### 2.2. Preparation of Ivermectin-Loaded Mesoporous Silica Particles

IVM was incorporated into commercial MCM-41 (hexagonal) nanostructured silica by the incipient wetness method [39]. For this, an ethanolic solution of IVM was prepared at a concentration of 17 mg/mL. Then, 700 μL of this solution was added dropwise to 100 mg of previously dried MCM-41. The sample was dried at room temperature until total evaporation of the solvent for 3 days. The mesoporous silica particles loaded with IVM were named IVM-MCM. The incipient wetness method was chosen considering the use of a low amount of organic solvent and the possibility of achieving high drug loading without significantly altering the physicochemical properties of the carrier material [39]. 

#### 2.2.1. Drug Content

The IVM content in the formulations was determined by high-performance liquid chromatography (HPLC) using a Shimadzu LC-System chromatography system (Kyoto, Japan). The parameters were co-validated based on a method described by Arantes (2011) [40], where the stationary phase was an end-capped Shim-pack ClC Shimadzu column (4.6 mm × 250 mm), whereas the mobile phase was composed of methanol:water (90:10), pumped at a flow rate of 1 mL/min. The injection volume was 50 μL, and IVM was detected at 254 nm. IVM-MCM was homogenized and dried for 1.5 h at 90 °C to remove moisture. Then, 8 mg of the sample was suspended in 50 mL of methanol. The system remained under ultrasonic agitation at room temperature for 2 h, followed by magnetic agitation for 1 h (750 rpm). At the end, the suspension was centrifuged at 4000 rpm for 6 min. The supernatant was collected, filtered (0.45 μL), and injected into the chromatographic system.

#### 2.2.2. Physicochemical Characterization

Thermogravimetric analysis (TGA) was used to determine the drug loading of the IVM-MCM and its thermal stability, using the Equipment Shimadzu Instrument model TGA-50 (Kyoto, Japan). The samples were heated from room temperature to 900 °C at a rate of 20 °C/min, under an argon flow of 50 mL/min. Differential Scanning Calorimetry (DSC) was performed to verify changes in the physicochemical properties of nanoencapsulated IVM in the MCM-41. The analyses were performed using the Shimadzu DSC-60 calorimeter (Kyoto, Japan) in the temperature range of 20–200 °C, at a heating rate of 10 °C/min, under a dynamic nitrogen atmosphere (50 mL/min). Samples MCM-41, IVM-MCM, IVM, and the physical mixture (drug:silica 0.12 mg/mg) were analyzed. X-ray diffraction (XRD) analyses were carried out to evaluate the crystallinity of IVM incorporated into mesoporous silica. The analyses were obtained using a diffractometer (Siemens X-ray Diffractometer—D-5000), equipped with a copper anode (CuKα). The samples (MCM-41, IVM-MCM, IVM, and physical mixture) were analyzed in an angle range of 2θ of 5–45°. In addition, low-angle XRD analysis (2θ of 1–7°) was performed to evaluate the pore organization of MCM-41. The textural characterization of nanostructured silica MCM-41, before and after the incorporation of IVM, was performed by nitrogen adsorption–desorption analysis (Tristar II Kr 3020, Micromeritics, Norcross, GA, USA). The samples were previously degassed at 60 °C for 12 h. The specific surface area was determined by the multipoint technique BET (Brunauer, Emmett, and Teller), and the pore size distribution was obtained by the BJH method (Barret, Joyner, and Halenda) [41,42].

### 2.3. Preparation of Ivermectin-Loaded Nanocapsules

IVM-loaded nanocapsules (IVM-NC) were prepared by the interfacial deposition of a preformed polymer method, following a protocol previously established by our research group [43]. An organic phase composed of IVM (1 mg/mL), 165 μL of medium-chain triglycerides, 0.1 g of poly(ε-caprolactone) (PCL), and 27 mL of acetone was completely homogenized under heating (40 °C) and magnetic stirring for 4 h. After that, injection of the organic phase was performed in the aqueous phase (54 mL) containing 0.077 g of polysorbate 80, whose resulting milky-like dispersion was kept under moderate agitation for 10 min. Afterward, acetone was removed, and the volume of water was reduced under low pressure to adjust the final volume to 10 mL containing 1 mg/mL of IVM. Triglycerides, particularly caprylic/capric triglyceride, were chosen as the oily core due to their excellent compatibility with PCL, being considered as a nonsolvent of the polymer [32].

#### 2.3.1. Physicochemical Characterization

The particle size distribution (D_[4,3]_) and polydispersity (Span) of IVM-NC were characterized by laser diffraction, without previous dilution of the samples (Mastersizer^®^, Malvern Instruments, Malvern, UK). In the sequence, analyses of z-average size and polydispersity index (PDI) were performed by the dynamic light scattering technique after dilution of samples (500×) in filtered (0.45 µm) ultrapure water, and zeta potential was measured using the electrophoretic mobility technique after dilution of samples (500×) in filtered (0.45 µm) 10 mM NaCl. These analyses were performed using Zetasizer^®^ equipment (Malvern Instruments, UK). The pH of the aqueous nanocapsules was evaluated with the aid of a previously calibrated potentiometer (DM-22—Digimed) without any previous dilution. 

#### 2.3.2. Drug Content and Encapsulation Efficiency

The drug content of the IVM-NC formulation was determined by HPLC according to the chromatographic conditions described in Section 2.2.1. Drug extraction from the organic nanoparticle procedure was carried out by adding 200 μL of the IVM-NC to 10 mL of methanol, followed by magnetic stirring for 6 min and vortexing for 2 min. Encapsulation efficiency (%EE) was determined after separation of IVM-NC from IVM free fraction by ultrafiltration–centrifugation at 4100× *g* for 10 min, and the drug amount was assayed by HPLC.

#### 2.3.3. Stability Study

The physicochemical stability of IVM-NC was evaluated at room temperature (~25 °C) in periods of 30, 60, and 90 days, following the assaying parameters described above. The formulations were prepared in three independent batches.

#### 2.3.4. Evaluation of the Presence of Nanocrystals

Most techniques for encapsulation efficiency assessment do not allow for differentiation of the presence of a drug in the form of nanoclusters or nanocrystals from those effectively encapsulated in the nanocarriers [44]. Their concomitant presence should be avoided in such types of formulations, as they can impair the physicochemical stability of the suspensions as well as change the in vitro drug release behavior, among others. In this evaluation, an IVM-NC formulation was divided into two bottles and stored protected from light. The first bottle was homogenized at the beginning of the experiment and before each sampling, whereas the second bottle was kept still during the entire study. The IVM content was determined in both samples after 15 and 30 days of storage.

### 2.4. Transmission Electron Microscopy (TEM)

The morphological characterization of the nanomaterials was performed by transmission electron microscopy (JEOL JEM-1011—Peabody, MA, USA) at an acceleration voltage of 100 kV, using carbon-coated copper grids. MCM-41 was suspended in isopropanol, with ultrasonic agitation for 5 min, and deposited in the grid. The IVM-NC suspension was diluted 1:10 (*v*/*v*) in filtered ultrapure water (0.45 μm) and deposited in the grid. As a negative control, uranyl acetate (2%, *w*/*v*) was used [45].

### 2.5. Ivermectin In Vitro Release Profile

The IVM release profile was evaluated using the diffusion method in a dialysis bag. One milliliter of IVM-NC was added to the dialysis bag, which was sealed and immersed in 150 mL of a dialysis medium, consisting of phosphate buffer solution (PBS) pH 7.2:ethanol (70:30 *v*/*v*%). For IVM-MCM, 8.17 mg of the sample was added to the dialysis bag and resuspended in 1 mL of PBS (≡1 mg/mL of IVM). For comparison purposes, free IVM (1 mg/mL) in ethanolic solution and aqueous dispersion in PBS (crystalline IVM) were simultaneously evaluated. The assay was performed in triplicate. The systems were maintained at 37 °C under continuous magnetic agitation. Sampling of the medium (1 mL) was conducted at predetermined times (0.5, 1, 2, 4, 8, 12, 24, 36, 48, and 72 h), and the same volume was replaced in the system. The system followed the sink condition, where the IVM saturation concentration was 1.13 mg/mL. IVM was assayed in the collected samples by HPLC, respecting the linearity range of 0.25 to 10 μg/mL.

### 2.6. Statistical Analysis

The statistical analysis of the results was delineated individually for each experimental protocol, demonstrated as the mean ± standard deviation when applicable. Data were analyzed for statistical significance through analysis of variance (ANOVA) using Dunnett, Tukey, and/or Bonferroni post-tests, where the statistical difference was considered significant when *p* < 0.05.

## 3. Results and Discussion

### 3.1. Ivermectin-Loaded Mesoporous Silica Particles

**Figure 2:**
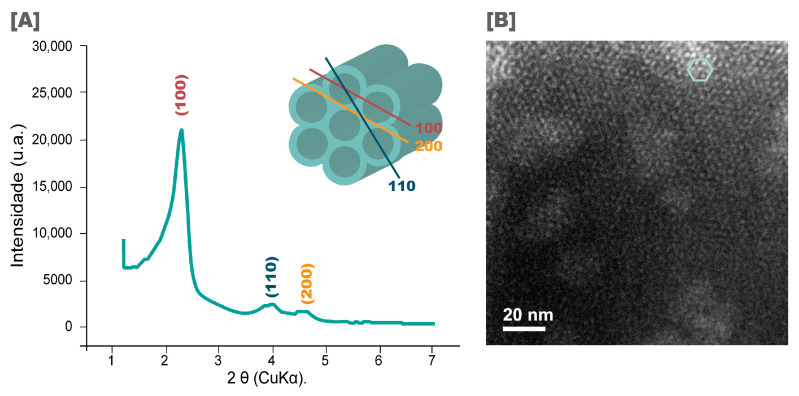
(**A**) X-ray diffractogram and (**B**) TEM image (magnification of 300,000×) of nanostructured silica (MCM-41).

Before drug loading, mesoporous silica particles (MCM-41) were characterized by XRD and TEM to evaluate their pore organization and morphology. The diffractogram for MCM-41 in the range of 2θ of 1–7° is shown in Figure 2A. As can be observed, there are peaks at 2θ = 2.3°, 3.9°, and 4.6°, respective to the planes at 100, 110, and 210, which are characteristic of MCM-41, proving the hexagonal arrangement of the mesopores [46]. This arrangement can also be observed in the TEM image shown in Figure 2B, where it is possible to see the regular organization.

**Figure 3:**
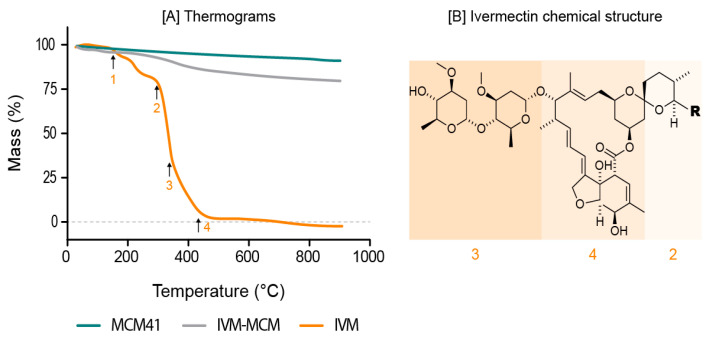
(**A**) Thermogravimetric analyses of nanostructured silica (MCM-41); ivermectin-loaded silica particles (IVM-MCM); and pure ivermectin (IVM). (**B**) Chemical structure of ivermectin, emphasizing the thermal decomposition stages.

Chromatography analysis (HPLC) demonstrated an experimental IVM content of 0.088 ± 0.007 mg/mg (>73% drug recovery). A recovery close to 70% could be related to the incomplete extraction of the drug from the silica particle, due to IVM adsorption forces on the silica pore surface [47]. Therefore, TG analysis was used as an additional method to evaluate drug loading in IVM-MCM. The thermal profiles of the MCM-41, IVM-MCM, and IVM samples were studied. The process of thermal decomposition of IVM occurs in four stages [48], according to the thermogram shown in Figure 3A, with the respective drug degradation processes indicated in Figure 3B. In the first region, around a temperature of 150 °C, the percentage mass loss is associated with the water desorption and alteration of the crystalline form of the drug. The second stage of thermal degradation occurs close to 300 °C and can be attributed to the degradation of the amphiphilic esters (C–O–C) of IVM due to the spatial configuration of the radical [49]. In the third region, at around 330 °C, molecular degradation begins, resulting in axial deformation of the O-H bonds and methyl groups, followed by unsaturated lactone breakage, between 350 and 400 °C. The total decomposition of the sample occurs above a temperature of 450 °C [49]. For MCM-41, the thermogram demonstrates a tiny decline in mass, indicating no organic residue in pure silica. The decline in the mass of silica is observed due to the process of dehydroxylation. This process involves the removal of hydroxyl groups (-OH) attached to silicon atoms, resulting in the release of water vapor and the formation of silanol groups (-Si-O-Si-) on the silica surface. The dehydroxylation of silica typically occurs in two stages: the removal of physically adsorbed water (around 50–150 °C) and the removal of chemically bound hydroxyl groups (above 600 °C) [50]. In the thermogram of IVM-MCM formulation, a decrease in mass was evident, in the region close to 400 °C, suggesting that the loss of mass is related to the decomposition of IVM.

Indeed, the nanostructured silica particles loaded with IVM (IVM-MCM) were produced with an estimated drug content of 0.12 mg/mg, representing a theoretical drug loading of 10.7% *w*/*w* in IVM-MCM. From the TGA data, the drug loading was calculated by subtracting the values of weight loss of MCM-41 from IVM-MCM in the range of 150 to 900 °C. The mass loss calculated by TGA was 9.98% *w*/*w* equivalent to a drug content of 0.11 mg/mg, which validates the theoretical amount of drug added at the beginning of the formulations, confirming that there was no substantial drug loss during the loading process (>90% drug recovery) and the hypothesis that part of IVM remains confined in the silica pores. 

**Figure 4:**
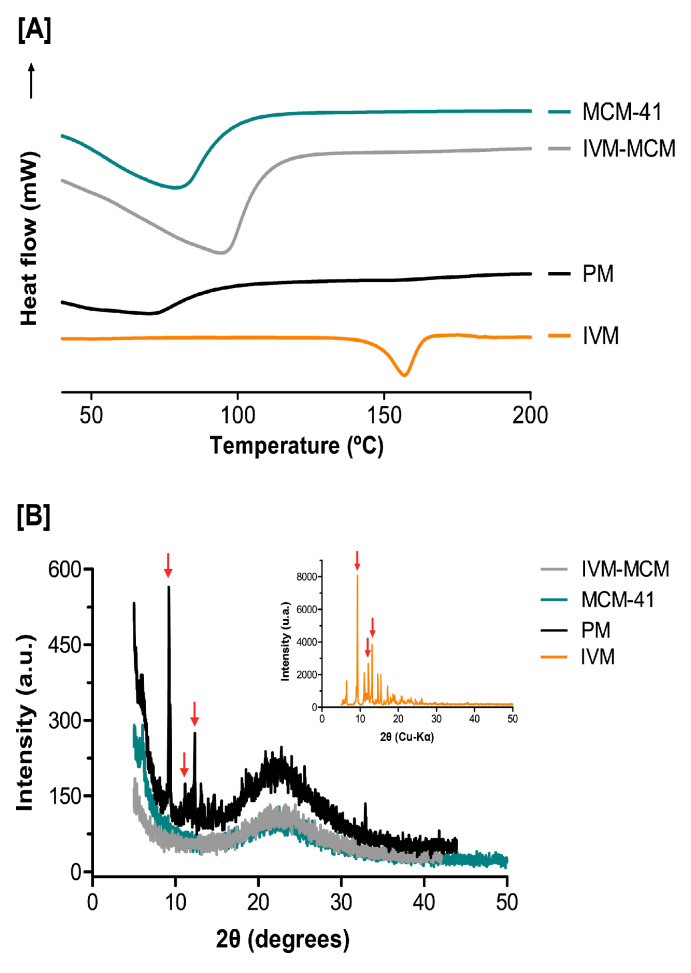
(**A**) Differential scanning calorimetry (DSC) and (**B**) X-ray diffraction (XRD) analyses of nanostructured silica (MCM-41); ivermectin-loaded silica particles (IVM-MCM); crystalline ivermectin (IVM); and physical mixture (MCM-41 + IVM). Red arrows show the crystalline IVM signal.

In addition, the DSC profiles (Figure 4A) revealed that IVM, after incorporation into the inorganic matrix, exhibited different thermal properties in comparison to its crystalline form. Indeed, the endothermic event resulting from the fusion of IVM at 155 °C [51], was not observed in the IVM-MCM samples. The absence of this endothermic peak of IVM in the IVM-MCM sample can indicate that the drug is highly dispersed in the pores of silica particles in an amorphous state. The lack of the crystalline IVM signal in the physical mixture (PM) curve may be due to the inherent sensitivity limitation of the DSC technique [37]. The crystalline signal of IVM might exist in extremely small quantities, potentially imperceptible. Thus, complementary thermal analysis by XRD was necessary, as this technique is highly sensitive to crystalline materials but significantly less sensitive to amorphous materials. DSC data were confirmed by XRD analyses (Figure 4B), where the IVM sample revealed the presence of a distinct peak at 2θ around 9.35° and secondary peaks of lower intensity at 11.08°, 12.1°, 13.15°, 14.66° and 15.44°, characteristic of the crystalline drug diffraction pattern [51], whereas MCM-41 alone did not show any diffraction pattern, only its characteristic amorphous halo in 2θ close to 22° [52]. On the other hand, the IVM crystalline pattern of reflections and the amorphous halo of silica appeared in the physical mixture of MCM-41 and IVM, while in IVM-MCM no IVM reflection were observed. The absence of the IVM diffraction pattern in IVM-MCM confirmed the high dispersion and amorphization of the drug inside the pores of silica particles, caused by confinement effects. Moreover, these data suggest that there was no drug overload in the silica: IVM ratio.

**Figure 5:**
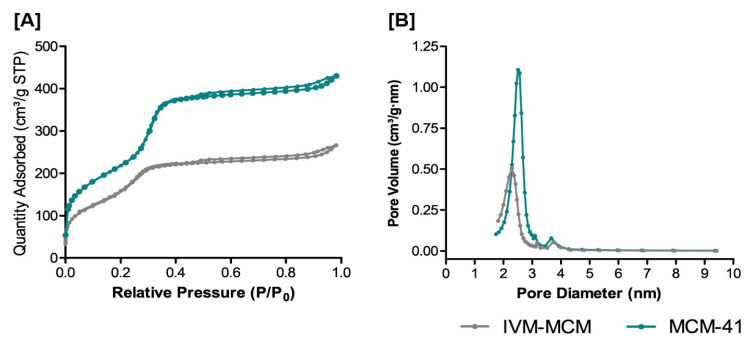
(**A**) Nitrogen adsorption–desorption isotherms and (**B**) pore size distributions for nanostructured silica (MCM-41) and ivermectin-loaded silica particles (IVM-MCM) samples.

Subsequently, to investigate the textural properties of MCM-41 and the effect of drug loading on the pore structure of IVM-MCM, and to confirm the amorphization by the drug confinement in mesoporous silica, N_2_ adsorption–desorption isotherms were acquired. Data are shown in Figure 5. The MCM-41 sample presented a surface area of 888 m^2^/g and a pore volume of 0.662 cm^3^/g (Figure 5A). The IVM-MCM samples demonstrated a reduction in this surface area and pore volume, reaching values of 672 m^2^/g and 0.394 cm^3^/g, respectively, which is in accordance with the pore-filling process. The maximum pore size distribution for MCM-41 was 2.5 nm, and a decrease in pore diameter was observed in IVM-MCM formulations with a shift of 0.21 nm in the distribution chart (Figure 5B). In addition, nitrogen intake in the pores of the IVM-MCM decreased, suggesting that the IVM is evenly distributed along the pore walls of the MCM-41. 

The findings support the successful adsorption and loading of IVM in an amorphous state onto mesoporous silica, resulting in high dispersion and a confinement effect on the drug. 

MSNs show promising properties as anthelmintic carriers, improving drug stability and release profiles. However, they have also been considered for delivering anthelmintics as repurposed cancer drugs due to their beneficial traits [52], such as biodegradability, biodistribution, controlled drug release, adjustable size, high drug loading, and the ability to transform drug crystalline structure, potentially enhancing solubility. An outstanding feature of MSNs is their high loading capacity compared to other nanoparticles. Additionally, they can gate cargo-loaded pores to reduce premature release, making them ideal for targeted delivery of therapeutic molecules [53]. Previous studies have demonstrated the high drug-loading efficiency of the avermectin class in MSNs. Polydopamine-modified mesoporous silica particles were investigated for drug release responsive to acidic environments [54]. Moreover, porous hollow silica nanoparticles loaded with avermectin and abamectin provided great stability with a strong protective effect on the loaded drug against photodegradation [55,56].

### 3.2. Ivermectin-Loaded Nanocapsules

**Figure 6:**
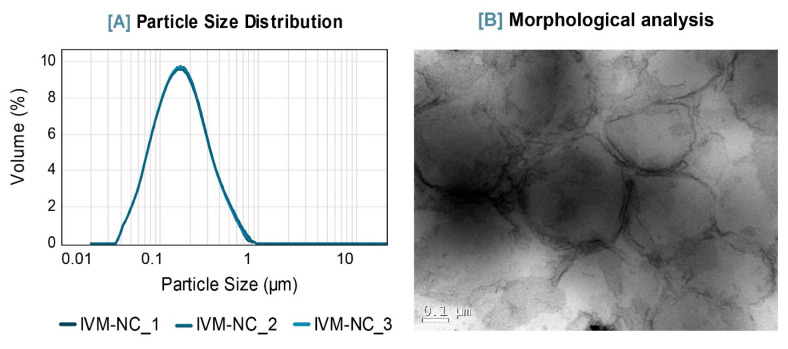
Physicochemical and morphological characterizations of IVM-NC. (**A**) Particle size distribution by laser diffraction analysis. (**B**) Morphological analysis by TEM. Image with a magnification of 200,000×.

IVM-NC were produced (n = 3) with a drug concentration of 1 mg/mL and had macroscopic characteristics of a homogeneous system, without precipitate formation. They showed a milky aspect and opalescence with a bluish reflex, related to the *Tyndall Effect*, indicating the presence of colloidal structures [57]. The aqueous suspensions had a slightly acidic pH of 4.8 ± 0.01. According to the laser diffraction analyses, they present a unimodal granulometric distribution exclusively in the nanometric range (Figure 6A), with a span of 1.8 ± 0.03 and a mean diameter size (D_[4,3]_) of 196 ± 2 nm. Polymeric nanoparticles usually have average diameters between 100 and 300 nm [58] and can vary according to the formulation constituents and the method of preparation. In order to refine the particle size data, dynamic light scattering analyses were carried out showing a Z-average of 202 ± 2 nm and a low polydispersity index (0.12 ± 0.01), which support the data obtained by laser diffraction. IVM-NC showed negative zeta potential (−17 ± 0.5 mV), suggesting a stable colloidal suspension [59] supported by the electronegativity of the compounds on their surface (PCL and polysorbate 80) as well as by the steric stabilization provided by the polysorbate 80. Figure 6B shows the TEM analysis of these nanoformulations, where spherical particles with regular and well-defined edges could be observed, whose sizes are consistent with the results obtained by the laser diffraction technique. 

The IVM content in the formulations was 1.00 ± 0.08 mg/mL, equivalent to 100% of the expected concentration but representing a low drug loading of 0.1% (*w*/*w*), markedly lower than the loading percentage encapsulated in the inorganic particles. On the other hand, the ultrafiltration–centrifugation assay indicated a high encapsulation efficiency of the IVM-NC (100%). This high encapsulation efficiency of IVM can be explained by its high log P, which favors its dispersion in the oily core of the nanocapsules and by its concentration below the drug saturation concentration in the oil. The influence of the presence of IVM nanocrystals on this encapsulation efficiency data was refuted, as no significant difference in content was observed between the static and agitated formulations, indicating no simultaneous formation of nanocrystals of the drug during nanocapsule preparation [60]. Due to the low drug loading, thermal analyses were not carried out for these formulations. The low drug content would hinder the visualization of relevant thermal events associated with the drug, potentially introducing bias into the subsequent analysis and discussion of the data. 

Lastly, physicochemical stability studies showed that IVM-NC suspensions are stable for 60 days if stored at room temperature (~25 °C) and protected from light (Table 1). They preserved their milky appearance, with no apparent changes in color, phase separation, or flocculation. The observed decrease in the value of zeta potential in the module may be due to polysorbate 80 auto-oxidation in the aqueous medium [61]. Similarly, the pH values also showed a decrease from 4.8 to 4.0 in a period of 30 days, which may also be related to oxidation products of polysorbate 80, such as acetic acid, formic acid, and non-ionic acids [61]. Despite these slight variations in pH and PZ of the IVM-NC over 60 days, the size distribution remained unimodal within the nanometric range during this period. On the other hand, reaching the period of 90 days of storage, the formulations presented some polymeric precipitates on their surface, culminating in a significant increase in particle size (615 ± 346 nm) and a loss of unimodal granulometric distribution (span > 2). This may be related to the hydrolysis of the polymer, poly(ε-caprolactone), as evidenced by the decrease in the pH value [45]. Therefore, for storage times longer than 60 days, it would be important to convert the liquid systems to solid intermediates using well-established techniques, such as spray-drying [62,63,64] or freeze-drying [65]. Something that should be highlighted in this discussion is the fundamental characterization of the particle size distribution by complementary techniques. As can be seen in Table 1, if only light scattering analyses had been carried out, the changes in the particle size would not have been detected. This data discussion is based on the particle size working range of each technique.

In summary, besides their lower drug loading compared to the silica mesoporous particles discussed earlier, IVM-loaded nanocapsules were produced with suitable nanotechnological properties. Their characterization data were consistent and reproducible as nanosystems for delivering IVM, in line with similar formulations reported in previous studies [43,66,67].

In terms of biological application, nanocapsules with smaller particle sizes (100 to 300 nm) have a larger surface area-to-volume ratio, which enhances their cellular uptake and penetration through biological barriers [29,30]. This increase can improve the delivery of IVM to target tissues or cells, enhancing its therapeutic efficacy. Furthermore, smaller particles tend to have prolonged circulation times due to reduced uptake by the reticuloendothelial system and escaping the rapid clearance by the mononuclear phagocyte system [30]. In addition, the surface charge of nanoparticles affects their stability, colloidal behavior, and interactions with biological components. Nanocapsules with a high zeta potential, ~30 mV (positive or negative), repulse each other, improving colloidal stability and reducing aggregation [63]. This stability is crucial for preventing premature drug release and ensuring uniform distribution in biological fluids, ultimately impacting the bioavailability and effectiveness of IVM. However, there is another approach to promote nanocapsule stabilization, based on the steric mechanism, usually using surfactants, such as polysorbate 80 or polyethylene glycol. This mechanism explains the physical stability of our formulation.

The pH directly influences the stability of the drug used and the biological tolerability of the formulation, making it an important criterion for future application of these formulations. For example, using the parenteral route, the pharmacopeia proposes a pH range of 6.0–9.0, while for topical application, a pH range between 4.5 and 6.0 is considered appropriate and a range of 4.0–5.0 is compatible with the oral route [62].

In the context of the development of a new formulation of IVM, achieving a narrow size range is crucial to influencing biodistribution and biological activity. The attractiveness of high encapsulation efficiency and stable physicochemical properties within nanocarriers is evident. Importantly, our results not only match but in some respects exceed those of previous studies reporting the IVM nanoencapsulation in organic delivery systems. For instance, an IVM formulation based in PCL nanocapsules containing a pumpkin seed oil core exhibited a size of approximately 400 nm, coupled with high encapsulation efficiency (98–100%) and good physicochemical stability (150 days of storage at 4 or 20 °C). The encapsulation efficiency of IVM within lipid nanocapsules exceeded 90%, exhibiting sustained physicochemical stability over 60 days of storage at 4 or 20 °C [13]. Furthermore, an inquiry by Ali and collaborators, focusing on poly(lactic-co-glycolic acid) (PLGA) nanoparticles containing IVM, revealed a uniform spherical morphology of nanoparticles with a diameter of 96 nm, accompanied by encapsulation efficiency > 70% [30]. Lastly, solid lipid nanoparticles (SLNs) containing IVM exhibited a spherical structure (150 to 400 nm), high encapsulation efficiency (98%), and physicochemical stability by 31 days of storage at 4 °C [29].

### 3.3. Ivermectin In Vitro Release Profile

**Figure 7:**
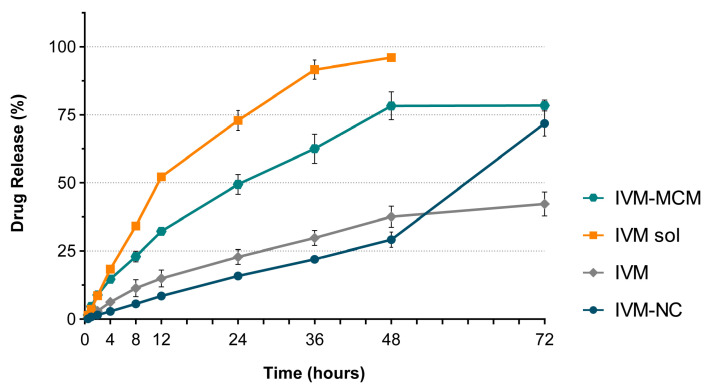
In vitro cumulative release of IVM from formulations of poly(ε-caprolactone) nanocapsule (IVM-NC) and nanostructured silica particles (IVM-MCM) compared non-encapsulated IVM (crystalline drug dispersion—IVM and drug ethanolic solution—IVM sol). The data are expressed as mean ± standard deviation (n = 3).

At the last stage of this study, the in vitro release/diffusion profiles of IVM encapsulated in mesoporous silica particles (IVM-MCM) and poly(ε-caprolactone) nanocapsules (IVM-NC) were evaluated by the dialysis bag method. The cumulative drug release in vitro was investigated over a period of 72 h, and the release profiles were compared with those of the crystalline IVM dispersion and IVM ethanolic solution, as shown in Figure 7.

**Figure 8:**
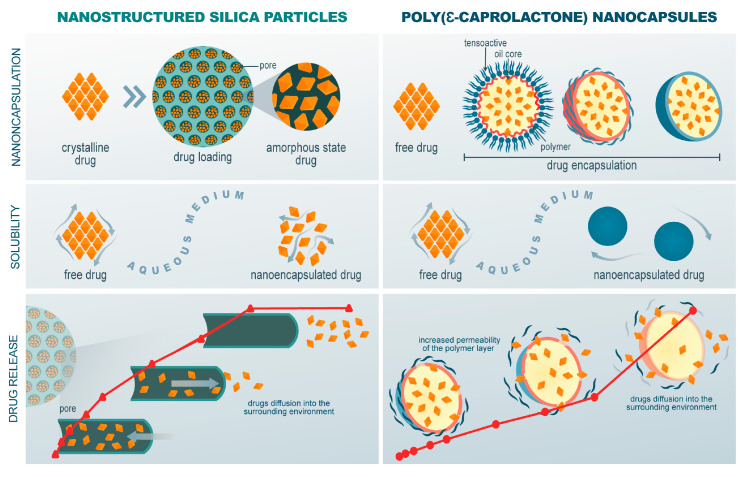
IVM nanoencapsulation of drugs, enhancement of drug solubility in aqueous medium, and drug release mechanism for poly(ε-caprolactone) nanocapsule and nanostructured silica particles.

A complete diffusion of IVM from an ethanolic solution (96 ± 2%) occurred after 48 h along with a similar release profile observed for the IVM-MCM (78 ± 3%). By contrast, crystalline IVM dispersed in an aqueous medium had very limited diffusion through the dialysis bag (35 ± 7%), most likely due to its low solubility in the aqueous medium and, consequently, low dissolution rate, as previously discussed for other poorly water-soluble drugs [38]. These results indicate an improvement in the apparent aqueous solubility of IVM when nanoencapsulated in the pores of MCM-41. On the other hand, the IVM-NC demonstrated gradual drug release over a period of 48 h, reaching cumulative IVM release of 29 ± 4%. While IVM-MCM showed a higher dissolution rate, IVM-NC presented a more controlled release, and this difference can be suggested to be due to the way the medium permeates each type of nanocarrier to dissolve the drug. The IVM molecules are adsorbed or loaded within the open pore structure of the silica particles. The drug release is diffusion-based, with the drug molecules moving through the pores and being released into the surrounding environment, as seen in the proposed mechanism represented in Figure 8 [68]. On the other hand, in polymeric nanocapsules, the IVM is encapsulated within the oily core of the nanocapsules. Therefore, drug release from nanocapsules occurs via drug diffusion through the semipermeable polymeric shell or by the dissolution or degradation of the polymer shell [69]. The release probably occurs because of the enhanced permeability of the polymeric layer, allowing the drug to be gradually diffused into the surrounding medium (Figure 8). This behavior is particularly evident in the time interval between 48 h and 72 h, where a significant steepening of the IVM-NC release curve is observed, attributed to the onset of increased water penetration through the polymer wall and subsequent drug diffusion. At 72 h, the IVM-NC reached a drug release of 72 ± 8%, achieving the cumulative drug release of IVM-MCM and surpassing the dissolution rate of the crystalline IVM.

From the data shown above, nanostructured silica particles and poly(ε-caprolactone) nanocapsules were able to increase the drug dissolution rate; however, this occurred in different chronologies. Both systems’ nanocarriers offer the potential for controlled and sustained drug release, but the underlying mechanisms rely on different principles based on the unique structures and the properties of each nanomaterial. The release rate of the drug from silica particles can be modulated by adjusting the pore size, surface functionalization, and interactions between the drug and the silica surface [68]. On the other hand, the release kinetics of polymeric nanocapsule release can be tailored by modifying the properties of the polymer shell, such as its thickness, composition, and charge. This provides control over the rate at which the drug is released and allows for sustained release over time.

IVM is highly lipophilic, with a water solubility close to 4 μg/mL [21] and a log P value of 5.83 [22]. The hydrophilic surfactant and polymeric shell of the nanocapsule stabilize the core and ensure the solubility of the drug in the aqueous medium. The characteristics observed in the release study, combined with previous results related to the high encapsulation efficiency of the drug, suggest that IVM may be retained within the oily nucleus of the nanocapsules, reflecting in a typical controlled release profile. Several studies have indicated that nanoencapsulation can effectively enhance the dissolution rate of hydrophobic drugs in water. For instance, the use of a poly(ε-caprolactone) nanocapsule with a copaiba oil core to encapsulate the hydrophobic anticancer drug imiquimod resulted in increased solubility and stability of the drug in an aqueous suspension [70,71]. Additionally, IVM-loaded solid lipid nanoparticles exhibited better stability and a sustained release profile in aqueous dispersion compared to a free drug suspension [29].

However, it is crucial to consider that the drug loading of nanoparticles can contribute to their drug release profile, aligning with Fick’s law of diffusion. This law establishes a direct relationship between the concentration of a substance and the speed of diffusion, stating that the rate of diffusion is proportional to the concentration gradient in the [72]. Applied to this study, the high drug load of IVM-MCM creates a greater concentration difference, imparting a faster diffusion rate.

As a different approach to increasing the aqueous solubility of lipophilic drugs, the confinement of the drug in nanometric pores after loading into mesoporous silica particles causes it to occur in a stable amorphous state [73]. The amorphous state of the drug provides an increased surface area, due to the spatial disorder of molecules, allowing greater mobility of the medium within the system and, consequently, reflects on increasing the dissolution rate of poorly soluble compounds in water (Figure 8) [74,75]. Moreover, a lower energy barrier is required to dissolve the amorphous drug compared to a crystalline drug because the crystal lattice disruption step does not need to occur [74].

The dissolution behaviors of drugs encapsulated in mesoporous silica particles have been previously documented by our group for triamcinolone acetonide loaded in SBA-15 silica. This study revealed a release profile identical to that of the drug in solution, providing evidence for the enhanced aqueous solubility of triamcinolone [37]. Similarly, the incorporation of clobetasol propionate in MCM-41 silica induced an alteration from a crystalline to an amorphous state, facilitating the drug dissolution process and subsequently increasing its apparent solubility [38].

As a conclusion of this study, both poly(ε-caprolactone) nanocapsules and nanostructured silica particles offer promising advantages in improving the dissolution rate of IVM and enabling sustained drug release. Poly(ε-caprolactone) nanocapsules demonstrate greater control over IVM release during the first half of the study, followed by a faster release towards the end. The combination of biodegradation targeted and/or passive delivery and controlled release achieved by polymeric nanocapsules could present an interesting strategy for the systemic administration of IVM. The polymer envelope acts as a protective barrier, safeguarding the encapsulated drug from degradation, enzymatic activity, and external factors until it reaches the desired site of action. On the other hand, these nanocarriers have a low drug loading capacity (less than 0.1% *w*/*w*).

By contrast, silica particles exhibit a support increased dissolution rate and constant IVM release throughout the study period with a high drug loading capacity (about 10% *w*/*w*). Their high surface area-to-volume ratio enables efficient adsorption of drug molecules and allows for quick penetration of the medium into the pores of the particle, resulting in faster but still sustained release. The ability to synthesize silica particles in various shapes and sizes offers flexibility for customizing drug loading and release profiles, and the possible surface functionalization can further enhance the stability, solubility, and targeting capabilities of the particle. Additionally, the thermal stability of silica particles makes them particularly interesting for veterinary applications, especially in topical use.

Therefore, considering the unique properties of each nanocarrier, the selection of the optimal option depends on specific application requirements, such as the type of payload, desired release kinetics, targeted cells or tissues, and compatibility with the biological system.

Finally, there are ongoing concerns about the adverse effects linked to the use of nanoparticles in clinical applications, highlighting the need for careful monitoring. Although previous studies have demonstrated the safe use of IVM-containing nanoformulations in cytotoxicity and in vivo toxicity assays [1], the evaluation of the potential toxicity of our specific formulation is essential in the near future. The unique properties of each nanomaterial have repercussions on specific interactions with the environment and biological components. Risks arise from the potential toxicity and interaction of nanoparticles with living cells. Attention should also be paid to potential changes induced by the biological media in nanoparticles, including chemical and physical alterations, and degradation, which affect their bioavailability and in vivo behavior [58].

## 4. Conclusions

In this study, we showcased an innovative approach to loading ivermectin (IVM) into silica mesoporous nanomaterial, achieving remarkably high drug loading and enabling drug amorphization. Additionally, we successfully produced IVM-loaded poly(ε-caprolactone) nanocapsules, featuring a uniform nanometric size distribution and high encapsulation efficiency, albeit with a lower drug loading capacity. Despite differing physicochemical properties, both nanocarriers exhibit promising advantages in improving the dissolution rate of IVM. The exploration of nanotechnology-based delivery systems emerges as a pivotal avenue for advancing novel approaches to administer poorly water-soluble drugs like IVM. Notably, this research represents a groundbreaking milestone as the first-ever report on employing silica nanomaterials as carriers for IVM delivery. Looking ahead, if we can surmount the biopharmaceutical challenges associated with IVM, these nanomaterials hold the potential to revolutionize the development of more efficient IVM formulations, addressing a diverse spectrum of diseases. This aligns with the overarching health needs of both humans and animals within the One Health framework. To delve deeper into the distinctive behaviors of these nanocarriers in pharmacological applications, further in vitro and in vivo studies using cancer models are on the horizon, paving the way for continued advancements in these drug delivery strategies.

## Figures and Tables

**Table 1:** Physicochemical stability of ivermectin-loaded nanocapsules stored at room temperature (~25 °C) for 3 months.

Physicochemical Parameters	Zero Time	30 Days	60 Days	90 Days
D_[4,3]_ (nm)	196 ± 2	214 ± 23	228 ± 8 *	615 ± 346 **
Span	1.38 ± 0.01	1.41 ± 0.07	1.51 ± 0.02	2.06 ± 0.47 *
Z-average (nm)	202 ± 2	200 ± 5	201 ± 8 *	198 ± 7
PDI	0.12 ± 0.01	0.09 ± 0.01	0.09 ± 0.04	0.08 ± 0.02
Zeta potential (mV)	−16.7 ± 0.5	−10.0 ± 1.6 **	−8.8 ± 0.7 **	−11.4 ± 0.9 **
pH	4.8 ± 0.1	4.0 ± 0.1 **	4.0 ± 0.1 **	3.9 ± 0.1 **
Drug content (mg/mL)	1.06 ± 0.08	0.96 ± 0.01	0.94 ± 0.02 *	0.97 ± 0.03

The results followed by an asterisk indicate the statistical difference when comparing the storage times at room temperature in relation to parameters of zero time, after preparation. The data followed by ** present statistically significant differences at *p* < 0.001 and * at *p* < 0.05, ANOVA followed by Dunnett’s test.

## Data Availability

The original contributions presented in the study are included in the article, further inquiries can be directed to the corresponding authors.

## Abbreviations

BCS	Biopharmaceutics Drug Classification System
DSC	differential scanning calorimetry
FDA	Food and Drug Administration
HPLC	high-performance liquid chromatography
IVM	ivermectin
IVM-MCM	ivermectin-loaded mesoporous silica particles
IVM-NC	ivermectin-loaded poly-ε-caprolactone nanocapsules
MCM-41	mesoporous silica particles type hexagonal
MSN	mesoporous silica nanoparticle
PBS	phosphate buffer solution
PCL	poly(ε-caprolactone)
PDI	polydispersity index
PLGA	poly(lactic-co-glycolic acid)
PM	physical mixture
SLN	solid lipid nanoparticles
TEM	transmission electron microscopy
TGA	thermogravimetric analysis
XRD	X-ray diffraction
